# Provoked overt recognition in acquired prosopagnosia using multiple different images of famous faces

**DOI:** 10.1080/02643294.2023.2269648

**Published:** 2024-01-12

**Authors:** David Pitcher, Rebekah Caulfield, A. Mike Burton

**Affiliations:** a Department of Psychology, University of York, York, UK; b Faculty of Society & Design, Bond University, Gold Coast, Australia

**Keywords:** Prosopagnosia, covert recognition, provoked overt recognition, face recognition

## Abstract

Provoked overt recognition refers to the fact that patients with acquired prosopagnosia can sometimes recognize faces when presented in arrays of individuals from the same category (e.g., actors or politicians). We ask whether a prosopagnosic patient might experience recognition when presented with multiple different images of the same face simultaneously. Over two sessions, patient Herschel, a 66-year-old British man with acquired prosopagnosia, viewed face images individually or in arrays. On several occasions he failed to recognize single photos of an individual but successfully identified that person when the same photos were presented together. For example, Herschel failed to recognize any individual images of King Charles or Paul McCartney but recognised both in arrays of the same photos. Like reports based on category membership, overt recognition was transient and inconsistent. These findings are discussed in terms of models of covert recognition, alongside more recent research on within-person variability for face perception.

## Introduction

Acquired prosopagnosia refers to the condition in which individuals experience deficits in face recognition, following brain injury. The underlying pathology generally involves damage to occipito-temporal and/or anterior temporal regions (Barton, 2008; Damasio et al., 1982; De Renzi et al., 1994). While prosopagnosia is often accompanied by other visual deficits, such as recognition of visual items with high inter-item similarity (De Haan et al., 1991), some patients have been reported with well-preserved object recognition abilities (McNeil & Warrington, 1993; Rezlescu et al., 2012; Rossion, 2022). Acquired prosopagnosia refers specifically to *faces*—patients are able to recognize familiar people from their voices or names, and often develop strategies, such as attention to people’s clothes, in order to navigate daily life.

In the 1980s, there were a series of reports demonstrating that some prosopagnosic patients retained *covert recognition* of familiar faces—that is to say, they showed some evidence of recognition when tested indirectly, but without any corresponding conscious experience. For example, when shown a series of faces, skin conductance measures were sensitive to the familiarity of the faces (Bauer, 1984; Tranel & Damasio, 1985). Covert recognition was also reported in diverse behavioural tests. When asked to learn face/name pairings, patients were faster to learn true pairs than false pairs (Bruyer et al., 1983; De Haan et al., 1987a; Sergent & Poncet, 1990). When classifying printed names (for example into categories such as “politician or popstar”?), reaction times were affected by face primes or face distractors (De Haan et al., 1987a; Young et al., 1988), despite no overt recognition of those faces.

Early enthusiasm for the study of covert recognition was based, partly, on the hope that it may offer some rehabilitative function. This prospect was boosted considerably by the first, very striking, report of provoked overt recognition by Sergent and Poncet (1990). Patient PV was densely prosopagnosic, but retained detailed knowledge about familiar people, and she showed evidence of covert recognition in the types of test described above. In order to try to prompt overt recognition, PV was presented with eight faces simultaneously and asked to name them—which she could not do. However, she was then told that the faces all belonged to people from the same profession. Following this prompt, PV was able to say that all were politicians, to name seven of them, and provide specific biographical details for the eighth. She reported that these were the first faces she had recognized in 15 years. This process was repeated with three more categories. For one of these (actors) PV was again able to name all eight faces, but for the other two categories (singers and TV personalities) she recognized none. Finally, faces were shown individually from all four categories, and PV was unable to recognize any of them, even those she had previously recognized.

Since the initial observation, there have been further reports of provoked recognition in prosopagnosia, all showing similar patterns: patients PH (De Haan et al., 1991), PC (Sergent & Signoret, 1992) and ET (Diamond et al., 1994) each recognized faces when presented together in categories, having failed to recognize the individual photos. However, each of these reports contained two aspects of PV’s performance which were not encouraging for rehabilitation. First, each of the patients only recognized faces in some of the categories presented. Second, the benefit of overt recognition was transient. Most patients failed to recognize individual photos almost immediately after having recognized them in a group. Patient PH did retain the ability to name individual faces when tested shortly after array presentation, but he recognized none of them when tested two months later. For these reasons, the study of provoked recognition was not pursued by those working in the field. For example, DeGutis et al. (2014) write “Though using covert recognition mechanisms to aid overt recognition is theoretically appealing and may be possible in particular situations for certain patients … . the findings have been too inconsistent to be useful for more general rehabilitation”.

Despite the overall loss of interest in covert recognition, we agree with De Gutis et al that the phenomenon is theoretically appealing. At the time of greatest activity in the field, there were two broad classes of account for covert recognition. First, some authors proposed that these patients retained an intact face recognition system, but this was disconnected from the processes that signal awareness (De Haan et al., 1987b; Tranel & Damasio, 1985). However, these disconnection accounts were severely challenged by the subsequent reports of provoked overt recognition. Instead, a second class of account became popular, proposing that the face recognition system was itself damaged, but retained enough residual processing to support covert, but not overt, recognition. These accounts were presented in terms of connectionist models which can be damaged in a way that produces degraded performance, without complete loss of function (Burton et al., 1991; Farah et al., 1993; Morrison et al., 2001; Young & Burton, 1999). Subsequent analyses of covert recognition have tended to retain this idea of a degraded face recognition system, but sought to further clarify how particular types of degradation might be linked to particular physiological and behavioural manifestations of covert recognition (Bobes et al., 2003; Schweinberger & Burton, 2003; Simon et al., 2011; Sperber & Spinnler, 2003).

In the years since covert recognition was widely studied, there have been some changes in focus for those studying face recognition more generally. In particular, it is becoming clearer that a key component of recognition involves within-person variability, i.e., the ways in which we can recognize multiple different photos as the same person, or “telling people together” (Burton, 2013; Jenkins et al., 2011). A strong marker of face familiarity seems to be its robustness to superficial image change (Johnston & Edmonds, 2009) and there is good evidence that people’s faces vary in *idiosyncratic* ways (Burton et al., 2016). This means that to become familiar with a face, a viewer needs to encode the range over which *that particular face* can vary. This suggestion has been supported by a number of studies reporting enhanced learning through exposure to variable images of the same face (Bindemann & Sandford, 2011; Matthews & Mondloch, 2018; Murphy et al., 2015; Ritchie & Burton, 2017) and by real face to face interactions (Sliwinska et al., 2022).

While the benefit of using multiple images for face learning is well-supported, there is less consensus for other tasks. In face *matching* tasks, such as checking photo-ID, viewers have to match a photo to a face presented concurrently. Some studies have been reported in which performance is improved by the use of multiple photos on the same “ID document” (White et al., 2014), whereas others have failed to observe this advantage (Ritchie et al., 2020). When searching real CCTV footage from a London transport hub, participants benefited from three photos of the target, but not more than three (Mileva & Burton, 2019). Ritchie et al. (2021) report consistent benefits for multiple photos in memory tasks, but not simultaneous matching tasks.

The recent interest in within person facial variability leads back to our earlier consideration of provoked overt recognition. It is interesting to ask whether a prosopagnosic patient might recognize multiple photos of the same person, when presented simultaneously. Theories of prosopagnosia emphasizing damaged, but not entirely eliminated, face processing, seem consistent with the possibility of provoked recognition in this case. However, the prediction is not trivial. Earlier reports of covert recognition relied on tests that showed similar patterns in prosopagnosic and neurotypical participants (e.g., priming, interference). There is no study of *recognition* using multiple photos of the same *familiar* face in typical viewers, for the simple reason that familiar face recognition is generally so good—participants are already at ceiling levels of recognition using a single photo, so there is no motivation to try to improve this. We have also noted above that not all tasks show a benefit for multiple photos. In particular, simultaneous matching tasks with unfamiliar faces do not consistently show an advantage for use of multiple images. Instead, the most reliable benefits of multiple image presentations seem apparent when abstractive processes are involved—i.e., in face memory or face learning. Here we ask whether presentation of multiple photos might somehow boost a damaged system to achieve overt recognition, albeit in a setting that does not involve learning.

In this paper, we report further experiments with the well-studied patient *Herschel*. A full case report can be found in Rezlescu et al. (2012), but we will summarize here. Herschel is a British male (born 1956), right-handed and educated to degree level. He had two strokes in 2008 causing damage to his occipitotemporal cortex (mostly right hemisphere) and right hippocampus. Herschel lost a large part of his upper visual field (extensively left, and a large part of the right). In two separate neuroimaging studies (performed eight years apart) scans were able to localize Herschel’s bilateral fusiform face area (FFA) and occipital face area (OFA), but the neural response to faces in all areas was impaired compared to age matched controls (Rezlescu et al., 2012; Sliwinska et al., 2020). He is densely prosopagnosic but shows normal ability in a wide range of object recognition tasks, including the ability to discriminate visually similar objects within category (Rezlescu et al., 2012). Herschel has also been shown to have normal levels of learning for visually similar objects, in the absence of parallel ability to learn new faces (Rezlescu et al., 2014). The study reported here was performed in 2022, when Herschel was aged 66, and 14 years after his stroke.

## Method

This study received ethical approval from the University of York Psychology Ethics Committee. Patient Herschel and all control participants gave informed consent to take part in the study.

### Overview

Herschel was tested on two occasions. In the first session he was shown a set of individual faces, one after another and asked whether he could identify any of the photos. These faces comprised 6 different photos of 10 famous individuals, randomly mixed with 6 different photos of each of 10 unfamiliar people. Following individual presentations, Herschel was shown arrays comprising the same 6 photos of each individual—all the people from the initial exposure phase—and again asked if he could recognize these people.

The second session followed a similar format, and took place 5 weeks later. Once again, Herschel saw individual faces of 20 people, 6 different photos of each, half famous. Some of these were the same people as in session 1 (details below). Following the sequence of individual faces, the patient was once again shown arrays of all 6 photos per identity. On this occasion he was then tested once again with the individual photos, in a different random sequence.

### Stimuli and context

It is useful to note that the UK was undergoing significant constitutional and political activity at the time of testing (Autumn 2022). Queen Elizabeth II had died four days before the first testing session, and was immediately succeeded by her son, King Charles III. A new prime minister (Liz Truss) had been appointed 6 days prior to the first testing session and was then forced to resign three days after the second testing. These events dominated media coverage in the UK, and many of the stimulus faces were chosen because they were key players.

Images of each of the famous people were derived from an internet search. The experimental procedure required a minimum of six images per person, though spares were also collected (see below). Images were cropped to remove background and any visible clothing but retained the hair. Once cropped, images were scaled to approximately 12 × 10 cm (recognition sequence stimuli); 6 × 5 cm (photo array stimuli) for presentation.

Famous faces for session 1 were: Bill Clinton, King Charles III, Paul McCartney, Margaret Thatcher, Ernie Wise, Jeremy Corbyn, Meghan Duchess of Sussex (aka Meghan Markle), Priti Patel, Sir Keir Starmer and Liz Truss. Note that the first five of these people were famous prior to Herschel’s stroke, whereas the last five had become famous only since then. This manipulation had no effect (see below) and so was not used as a criterion for selecting faces for session 2. Three of the session 1 target people were replaced for session 2 because Heschel had recognized none of their photos in either individual or array presentations (Wise, Corbyn, Markle). To balance male and female targets, we also replaced Bill Clinton with Hillary Clinton. The faces in session 2 were: Hillary Clinton, King Charles III, Paul McCartney Margaret Thatcher, Sean Connery, George Clooney, Priti Patel, Catherine Princess of Wales (aka Kate Middleton), Sir Keir Starmer and Liz Truss.

Images of unfamiliar people used as control stimuli were foreign celebrities, very unlikely to be known in the UK. These were Annalena Baerbock (German Politician), David Bisbal (Spanish Singer), Carmen Calvo (Spanish Politician), Charlene de Carvalho-Heineken (Dutch Businesswoman), Jason Clare (Australian Politician), Haakon Magnus (Crown Prince of Norway), Jesse Klaver (Dutch Politician), Sebastian Kurz (Austrian Politician), Johanne Schmidt-Nielsen (Danish Politician), Nikos Vertis (Greek Singer).

### Procedure

*Session 1*: Testing took place on-line, using Zoom. Herschel was initially briefed as to the nature of the task. It was explained to him that he would see a sequence of individual photos, and he would be asked to say if he recognized each photo, and if so whether he could provide any information about that person, including name, occupation, and any other information that came to mind. Each photo remained on the screen for 3s, and the response period was self-paced, allowing Herschel to respond to the above questions.

There followed a rest period of 5 min, after which Herschel was shown a sequence of photo arrays, each comprising six images of the faces seen earlier. The same photos were used as in the earlier individual presentation, except in cases that Herschel had recognized a photo. In this case, the experimenter substituted the recognized photo with a spare. So, none of the arrays contained a previously-recognized image.

For each of these arrays, Herschel was asked the following four questions in sequence, with an opportunity to respond to each question before moving to the next: “Do you recognise any of these people?”; “They are all the same person, do you know who?”; “Can you give any information about the person?”; “Which of these images do you recognise?”. Arrays remained on screen until Herschel had responded, and the whole session lasted 60 min.

*Session 2*: The format of the second session was identical to the first, with the addition of a further test of individual photo recognition at the end of the session. The whole session lasted 75 min.

Finally, to confirm that the familiar people used in this study were familiar to Herschel, he was asked to provide information to their written names. This test was performed four weeks after the completion of Session 2.

*Control participants:* Ten age-matched controls were recruited to check the familiarity and recognisability of the famous face images. These were volunteer participants, three women and seven men, with no reported neuropsychological history. Mean age was 67.5 (sd = 5.5). Testing took place online, using Zoom. Participants were presented with each of the individual images seen by Herschel in sessions 1 and 2. As above, images were presented for 3s, and participants were asked to say whether they recognized each photo, and if so whether they could provide any information about that person, including name, occupation, and any other information that came to mind.

## Results

In the test of written names, completed four weeks after the second face test, Herschel successfully recognized all names and was able to give biographical details about all the famous individuals from both experimental sessions. In many cases, he was expansive and wry in his descriptions, providing convincing evidence that he was familiar with each of the famous individuals used here.

Table 1 shows session 1 face recognition performance by Herschel and summary data for controls. Since we are interested in recognition of specific famous individuals, we present data for each face. Herschel recognized some of the individual photos, and for all these faces he also recognized the arrays. However, he failed to recognize any individual photos for 6 of the 10 famous people. For three of these (King Charles, Paul McCartney and Priti Patel), he went on correctly to identify the people when all their face images were presented in an array. Herschel made no false positive responses, i.e., he responded “unfamiliar” to all the non-famous faces, whether presented separately on in arrays. In contrast, controls did make a small number of false positive errors (mean = 5%, sd = 0.08).

**Table 1.** Face recognition rates for Herschel and controls, session 1.

	Herschel			Controls	
	Individual photos (/6)	Array		Mean (/6)	SD
Famous pre-2008					
Bill Clinton	3	Correct		6	0
King Charles III	0	Correct		6	0
Paul McCartney*	0	Correct		6	0
Margaret Thatcher	5	Correct		6	0
Ernie Wise	0	No ID		5.2	.87
Famous post 2008					
Jeremy Corbyn	0	No ID		6	0
Meghan Markle	0	No ID		6	0
Priti Patel	0	Correct		5.3	1.79
Sir Keir Starmer	2	Correct		6	0
Liz Truss	2	Correct		6	0

Note: (*Only 5 individual photos of Paul McCartney were shown, due to computer error).

Table 2 shows session 2 face recognition performance by Herschel and controls. Once again, Herschel recognized some individual photos, though there was little consistency across sessions. He also displayed provoked overt recognition for one of the faces, Paul McCartney, for whom Herschel recognized no individual photos on first presentation, but then did recognize the person when shown an array of all the photos. As with session 1, Herschel made no false positive responses to non-famous faces.

**Table 2.** Face recognition rates for Herschel in session 2.

Stimuli	Individual photos (/6)	Array	Individual photos 2nd run (/6)	Controls mean(sd)
King Charles III	2	Correct	2	6 (0)
Hilary Clinton	0	No ID	0	5.6 (.66)
George Clooney	0	No ID	0	6 (0)
Sean Connery	0	No ID	0	5.8 (.4)
Paul McCartney	0	Correct	1	6 (0)
Catherine Princess of Wales	0	No ID	0	5.7 (.64)
Priti Patel	0	No ID	0	5.3 (1.79)
Sir Keir Starmer	5	Correct	2	6 (0)
Margaret Thatcher	1	Correct	2	6 (0)
Liz Truss	3	Correct	2	6 (0)

Finally, in trials when Herschel was able successfully to identify a face we asked him what visual aspects he had used to recognize that individual. His insights tended to emphasize idiosyncratic physical attributes of the people depicted. For example, after recognizing the leader of the UK Labour party Herschel reported “I think it may be from seeing a lot of Keir Starmer on TV and recognising him from the way he positions his mouth”. When presented with images of Margaret Thatcher, whom he had met as a schoolchild, he reported “there was a way she would hang her head … . at a slight angle” and that she had a “glaring look when hectoring someone, something only people of my age would recall”.

## Discussion

The data presented here show provoked overt recognition using multiple images of the same person. On several occasions (4 across the two sessions), Herschel failed to recognize any individual photos of a particular person, but went on to recognize that person when shown these same images simultaneously in an array. This pattern seems rather similar to provoked overt recognition by occupational category, as described in the earlier literature (see Introduction). However, this is the first time, to our knowledge, that multiple images of the same person have been used to elicit recognition in a patient with prosopagnosia. Before discussing possible underlying mechanisms for this effect, we first consider some of the detailed aspects of the data. Taken together, these are important for understanding the phenomenon of provoked recognition.

1. Like many acquired prosopagnosic patients, Herschel does sometimes recognize an individual photo. However, presenting multiple photos of the same person increased the likelihood of recognition. Of the 7 arrays recognized in session 1, Herschel had recognized some individual photos for four of those people, but not the remaining three. There were no cases in which he recognized an individual photo but failed to recognize an array for that person, even though his performance was inconsistent within and across sessions. The evidence is consistent with an advantage for array presentation, rather than random fluctuation in responses.
2. The pattern of responses suggests that Herschel’s face recognition system is damaged, but not entirely eliminated. However, his performance is severely impaired and inconsistent, and is not explained by any simple factor, such as “degree of familiarity”. For example, in session 1 he failed to recognize King Charles, even though he had recently acceded to the throne, and was very widely featured in the media at the time. Likewise, he failed to recognize Paul McCartney, on either occasion. He recognized 5 individual photos of Margaret Thatcher in session 1, but only one of these same photos in session 2. This pattern suggests a damaged and somewhat unstable recognition system.
3. Session 1 demonstrates that Herschel does not only recognize those people he knew before his stroke. He was able to recognize individual photos of two people famous before his prosopagnosia, and two people who became famous afterwards. Once again, this suggests a damaged, but not eliminated face recognition system, capable of acquiring some new representations post injury.
4. Provoked overt recognition was observed for only some of the individual faces. This pattern is very similar to the literature on provoked recognition by category, reviewed above. In those cases, patients always showed overt recognition for some categories but not others. We also note that Herschel was not consistent between sessions: arrays provoked overt recognition of Priti Patel in session 1, but not session 2. In contrast, Paul McCartney’s array provoked recognition in both sessions.
5. There is no consistent benefit of provoked recognition across sessions. Individual images of King Charles were recognized in session 2, having been provoked by an array in session 1. However, successful array recognition in session 1 did not lead to individual photo recognition of Paul McCartney or Priti Patel in session 2.
6. There is no consistent effect of repeated presentations, and so the benefit of the array cannot be due to the fact that arrays were always presented after an individual photo phase. In study 2, individual photos were presented twice, with the array task intervening. Recognition of the individual photos improved between runs for two items (McCartney and Thatcher), got worse for two items (Starmer and Truss) and remained the same for one item (Charles). There is therefore no hint of a general improvement with exposure.
7. Differences in timing between conditions seem unlikely to contribute to this effect. Array presentations were not time limited, to allow for increasingly specific questions to be asked (see above). However, Herschel was relatively quick to answer in response to correctly-identified arrays—for example for the 7 identified arrays in session 1, his mean voice onset time from array presentation was 6 sec (sd = 2.6) and this time included the experimenter asking the first question: Do you recognize any of these people?

How is it possible to explain the pattern of recognition exhibited by Herschel? We propose that these data are remarkably consistent with previous reports of provoked overt recognition, based on presenting people from the same category (e.g., politicians or actors). The most satisfactory theoretical accounts of those cases relied on a damaged, but not destroyed, face recognition systems. Patients appeared capable of some information processing, but not enough to support reliable overt recognition. This seems to be consistent with MRI evidence from Herschel, showing identifiable FFA and OFA responses, which were nevertheless weaker than those of controls (Rezlescu et al., 2012; Sliwinska et al., 2020). Different computational models have demonstrated that such a system can, under certain circumstances, acquire activation from multiple simultaneously presented sources, sufficient to trigger recognition (Farah et al., 1993; Morrison et al., 2001; Young & Burton, 1999). In principle, this same argument applies to the present demonstration.

One way to conceive the recognition process is based on the IAC model of face processing (Burton, Bruce, et al., 1999). Following Bruce and Young (1986), this model proposes “face recognition units”, visual representations that become active on exposure to any recognizable photo of a particular face. Such units take their input from early visual processing. It is relatively simple to conceive of inputs from different photos of the same face passing activation to the relevant FRU in slightly different ways—i.e., one photo might have a particularly salient (and person-relevant) hairstyle, another might have a characteristic expression. We propose that an FRU receiving input from multiple photos simultaneously may achieve levels of activation higher than would be the case for a single input image—a proposal that seems consistent with Herschel’s own insights about those images he recognizes. This results in greater activation through the system, increasing the likelihood of accurate recognition.

This way of thinking about provoked recognition highlights the remarkable ability of typical viewers to recognize familiar faces. Most people are able to recognize the faces they know in a very wide range of presentations, including highly impoverished images (Burton, Wilson, et al., 1999). This has tended to obscure the importance of “telling people together” in the literature, and the importance of within-person variability only becomes evident for typical viewers when processing unfamiliar faces, which are much more difficult to identify (Jenkins et al., 2011). The patient Herschel, unlike typical viewers, is very far from ceiling performance on recognizing familiar faces, and this provides the opportunity to study the contribution of within-face variability to recognition. An array contains more information about a person than a single photo, and equally importantly it contains information about what aspects of the images are not diagnostic of identity, i.e., those aspects that change from photo to photo. In Heschel, we can see the benefit of this information directly on recognition, a benefit which can only be tested indirectly, with unfamiliar faces, for typical viewers.

While provoked overt recognition remains an interesting phenomenon for understanding the face recognition system, there is unfortunately nothing in our data to suggest it might be useful for future rehabilitative programmes. Just as with categorically-provoked recognition, the effect described here occurs only sometimes (i.e., for some faces), is not stable between testing sessions, and appears to be transient. Unfortunately, the most useful interventions for acquired prosopagnosia still seem to be based on compensatory training, for example emphasising piecemeal facial features or non-facial identity cues (DeGutis et al., 2014).

In recent years, there has been a growing interest in developmental prosopagnosia, i.e., face recognition deficits not linked to specific insult or injury (Behrmann & Avidan, 2005; Dobel et al., 2007). Contemporary analyses seek to understand the relationship between acquired and developmental forms, though both conditions admit a very broad range of deficit patterns (Corrow et al., 2016; White & Burton, 2022). It would be especially interesting to establish whether the provoked recognition phenomenon described here may also be observed in developmental prosopagnosia (DP). There are reasons to predict that it might. There is good evidence for covert recognition in people with DP across a number of different tasks (Avidan & Behrmann, 2008; Eimer et al., 2012; Rivolta et al., 2012). Interestingly, Rivolta et al. (2012) propose that some tasks that apparently fail to demonstrate covert recognition in DPs might actually be better measures of provoked overt recognition—opening up the possibility of testing for this, even though it has not previously been observed.

Techniques developed more recently for research on within-face variability may also be useful here. For example, typical viewers are poor at sorting images of unfamiliar faces into their identities—often believing there are many more people present than there actually are (Baker et al., 2017; Jenkins et al., 2011). However, when explicitly told how many people are present—normally just two—participants are very good at the sorting task (Andrews et al., 2015). The constraint of knowing that faces can only be grouped into a small number of categories appears to support coherence of multiple images into a single representation. Whether this would be helpful in developmental prosopagnosia remains an interesting question.

In summary, we have presented the first demonstration of provoked overt recognition in acquired prosopagnosia, using multiple images of the same face. The pattern of results is consistent with previous reports of categorically induced provoked recognition, and is also consistent with recent findings showing the importance of within-person variability for familiar face recognition. Consistent with earlier findings, the relief from prosopagnosia is temporary and inconsistent, but nonetheless compelling.
